# Valorization of Char From Biomass Gasification as Catalyst Support in Dry Reforming of Methane

**DOI:** 10.3389/fchem.2019.00119

**Published:** 2019-03-13

**Authors:** Vittoria Benedetti, Snehesh Shivananda Ail, Francesco Patuzzi, Marco Baratieri

**Affiliations:** Faculty of Science and Technology, Free University of Bolzano, Bolzano, Italy

**Keywords:** char, biomass gasification, dry reforming of methane, catalyst, cobalt, magnesium oxide

## Abstract

This study responds to the need of finding innovative routes for valorizing char derived from biomass gasification. Char is currently treated as a waste representing an energetic and economic loss for plant owners. However, it displays many similarities to activated carbon (AC) and could replace it in several applications. In this regard, the current work investigates the use of gasification derived char as catalyst support in dry reforming of methane (DRM) reactions. Char collected from a commercial biomass gasifier currently in operation was characterized and employed for the synthesis of cobalt catalysts. The catalysts were characterized and tested in an atmospheric pressure fixed bed reactor operating at 850°C with CH_4_:CO_2_ = 1 and a weight hourly space velocity of 6,500 mL g^−1^ h^−1^. The effectiveness of the synthesized catalysts was defined based on CO_2_ and CH_4_ conversions, the corresponding H_2_ and CO yields and their stability. Accordingly, catalysts were synthesized with cobalt loading of 10, 15 and 20 wt.% on untreated and HNO_3_ treated char, and the catalyst with optimum comparative performance was promoted with 2 wt.%MgO. Catalysts prepared using untreated char showed low average conversions of 23 and 17% for CO_2_ and CH_4_, yields of 1 and 14% for H_2_ and CO, and deactivated after few minutes of operation. Higher metal loadings corresponded to lower conversion and yields. Although HNO_3_ treatment slightly increased conversions and yields and enhanced the stability of the catalyst, the catalyst deactivated again after few minutes. On the contrary, MgO addition boosted the catalyst performances leading to conversions (95 and 94% for CO_2_ and CH_4_) and yields (44 and 53% for H_2_ and CO) similar to what obtained using conventional supports such as Al_2_O_3_. Moreover, MgO catalysts proved to be very stable during the whole duration of the test.

## Introduction

Amongst the thermochemical technologies for processing lignocellulosic biomass into bioenergy and biofuels, biomass gasification stands out for the high conversion efficiencies achievable, the low emissions and the ease of their control, the flexibility of feedstocks in input and the range of output products. The main purpose of biomass gasification is the production of a gaseous mixture of CO, H_2_, and CH_4_, to be used in combined heat and power (CHP) engines for the cogeneration of heat and electricity or for the production of chemicals and fuels (e.g., F-T diesel, methanol, hydrogen, etc.; Basu, 2010). However, along with gas, by-products are formed, namely tar and char. Tar is a black bituminous viscous liquid, highly undesirable due to its tendency to condense in the low-temperature zones of a gasifier, clog the pipes and downstream equipment (Basu, 2010). On the other hand, char is a solid carbonaceous material that accounts for nearly 10% of the original feedstock and it is currently treated as an industrial waste (Benedetti et al., 2018).

This work considers the Italian region of South Tyrol as a benchmark. Annually, 1,300 tons of char is produced over the entire region (Basso et al., 2018). The substantial char yield is an outcome of 46 small-scale gasification plants, with an average electrical output ranging from 25 to 440 kW and operational as on 2018. The associated disposal cost of char ranges from 140 to 150 €/ton (Patuzzi et al., 2016).

To avoid the economic losses for biomass plant operators and avert the environmental issues related to its disposal, it is crucial to find innovative solutions for char valorization. The growing interest in this issue, has led the scientific community to tentatively employ char for combustion (Galhetas et al., 2012), gas and dye adsorption (Runtti et al., 2014), catalyst preparation (Ahmad et al., 2018), tar cracking (Klinghoffer et al., 2012), soil fertilization (Hansen et al., 2015).

Our previous studies showed that char collected from commercial biomass gasifiers, have many similarities with activated carbon (AC) with respect to its physical and chemical properties (Benedetti et al., 2018). Similar to AC, char displays a high carbon content up to 90%, a high surface reactivity, a large specific surface area of ~600 m^2^/g and a well-developed microporosity (Marsh and Rodriguez-Reinoso, 2006). Consequently, due to its remarkable properties, char could substitute AC in several applications.

Activated carbon and other carbon-based materials such as carbon nanotubes, carbon spheres, carbon rods and ordered mesoporous carbon have been widely used in the field of heterogeneous catalysis. As catalyst supports, they offer several advantages like high surface area, versatile and adjustable pore size distribution and surface functional groups, reductive properties and the possibility of recover the active metals from the deactivated catalysts by burning off the carbonaceous matrix (Serp and Figueiredo, 2009). Moreover, it has been observed that an inert material like carbon can reduce the metal-support interactions that are predominantly present in the conventional supports (e.g., Al_2_O_3_, SiO_2_, TiO_2_), resulting in a potential increase of the catalytic conversion efficiencies (Ail and Dasappa, 2016). Considering the physicochemical similarities of gasification char and AC, also char could be included in this wide spectrum of materials. In this regard, the current study investigates the feasibility of using char as catalyst support in dry reforming of methane (DRM), i.e., the catalytic conversion of a mixture of CO_2_ and CH_4_ into syngas, H_2_ and CO.

DRM was selected as test reaction because of its potential benefits on consuming greenhouse gasses (GHG) like CO_2_ and CH_4_ while producing useful chemical building blocks for downstream industrial processes as methanol, dimethyl-ether or Fischer-Tropsch synthesis (Gao et al., 2011). Thanks to this process, CO_2_ emissions are reduced to around 0.2 m^3^ of CO_2_ per m^3^ of H_2_ produced, high purity CO is generated and H_2_/CO can be manipulated to obtain optimal ratios suitable for further applications (Arora and Prasad, 2016). Moreover, DRM could be also applied to biogas from anaerobic digestion, a gas mixture typically composed by CH_4_ (55–65%), CO_2_ (30–45%), and H_2_S (0.5–2%), after proper drying and H_2_S scrubbing (Chattanathan et al., 2014). The aim of the coupling is two-fold: reducing the GHG emissions associated with the process, and providing a renewable source of hydrogen (Lavoie, 2014).

Furthermore, in recent years, innovative processes, such as chemical looping dry reforming (CLDR), have been studied and coupled to DRM in order to exploit biogas from anaerobic digestion in a more efficient way, avoid some of the criticalities of the DRM process and reduce CO_2_ emissions more effectively (Mendiara et al., 2018). DRM entails several reversible reactions comprising CO_2_ and steam reforming (R.1 and R.2), CO_2_ methanation and reverse water gas shift reaction (R.3 and R.4), methane cracking (R.5), Bouduard reaction (R.6), carbon gasification (R.7), and CO methanation (R.8) (Haghighi et al., 2007; Nikoo and Amin, 2011).

$$\begin{array}{l} \begin{array}{llll} {\text{CH}}_{4}+{\text{CO}}_{2}↔2\text{CO}+2{\text{H}}_{2} & \Delta {\text{H}}_{298}=+247\text{kJ}/\text{mol} & {\text{CO}}_{2}\text{ reforming} & \left(R.1\right)\\ {\text{CH}}_{4}+{\text{H}}_{2}\text{O}↔3{\text{H}}_{2}+\text{CO} & \Delta {\text{H}}_{298}=+206\text{kJ}/\text{mol} & \text{Steam reforming} & \left(R.2\right)\\ \text{C}{\text{O}}_{2}+4{\text{H}}_{2}↔\text{C}{\text{H}}_{4}+2{\text{H}}_{2}\text{O} & \Delta {\text{H}}_{298}=-165\text{kJ}/\text{mol} & \text{C}{\text{O}}_{2}\text{ methanation} & \left(\text{R}.3\right)\\ \text{C}{\text{O}}_{2}+{\text{H}}_{2}↔\text{CO}+{\text{H}}_{2}\text{O} & \Delta {\text{H}}_{298}=+41\text{kJ}/\text{mol} & \text{Reverse water gas shift} & \left(R.4\right)\\ \text{C}{\text{H}}_{4}={\text{C}}_{\left(\text{s}\right)}↔2{\text{H}}_{2} & \Delta {\text{H}}_{298}=+75\text{kJ}/\text{mol} & \text{Methane cracking} & \left(R.5\right)\\ 2\text{CO}↔{\text{C}}_{\left(\text{s}\right)}+\text{C}{\text{O}}_{2} & \Delta {\text{H}}_{298}=-171\text{kJ}/\text{mol} & \text{Bouduard reaction} & \left(R.6\right)\\ {\text{H}}_{2}\text{O}+{\text{C}}_{\left(\text{s}\right)}↔\text{CO}+{\text{H}}_{2} & \Delta {\text{H}}_{298}=+131\text{kJ}/\text{mol} & \text{Carbon gasification} & \left(R.7\right)\\ \text{CO}+3{\text{H}}_{2}↔\text{C}{\text{H}}_{4}+{\text{H}}_{2}\text{O} & \Delta {\text{H}}_{298}=-206\text{kJ}/\text{mol} & \text{CO methanation} & \left(R.8\right) \end{array} \end{array}$$

DRM is a highly endothermic process occurring at temperature higher than 800°C. Nearly 30% or more of the methane supplied must be used to satisfy the high energy requirements (Yu et al., 2017). However, if this energy is provided by the combustion of fossil fuels, further CO_2_ emissions will be unavoidable and the main environmental benefits of the process will be undermined. Therefore, if renewable energy sources were used for feeding the process, DRM would be beneficial not only in terms of CO_2_ reductions, but also in providing strategies to their storage and transport (Edwards and Maitra, 1995). In fact, the intermittent and unstable nature of renewable energy sources and their low energy density are main obstacles for their industrial utilization in large-scale heat and power applications (Chen et al., 2018). DRM could be a suitable form of thermo-chemical storage to efficiently store excess energy in a chemical form, at ambient temperature, especially when solar energy is considered (Tavasoli and Ozin, 2018).

Methane molecules dissociation is favored not only at high temperature but also in presence of appropriate catalysts. Catalysts play a fundamental role in any Power-to-X (PtX) technology and the selection of a valid catalyst is crucial for the efficiency and economy of the process.

Coal char itself was reported as a promising catalyst for DRM (Muradov et al., 2005). However, the associated CH_4_ and CO_2_ conversions are much lower than those of metal-based catalysts operating under similar reaction conditions, especially at temperatures below 900°C.

Transition metals of group VII, IX, and X catalyze the DRM reaction and although highest activity is reported by noble metal based catalysts, Ni is usually employed owing to its lower cost and better availability (Lavoie, 2014). However, its tendency toward carbon deposition hampers elongated performances and provides undesirable deactivation phenomena. Carbon deposition is more likely to occur when the generation of carbon species through methane cracking (R.5) is faster than the rate of carbon removal by gasification reactions (R.6–7) (Haghighi et al., 2007).

Recently, many studies have focused on the use of cobalt as active metal for DRM (Budiman et al., 2012). Even though the catalytic activity of Co-based catalysts is not superior to the Ni-based catalysts, the former show better stability and slower deactivation, probably due to the different mechanisms of carbon deposition involved. Hence, in this study cobalt was selected as active metal for the synthesis of char-supported catalysts.

The possibility to combine the catalytic effects of both carbon-based supports and active metals is very appealing.

Several studies in the literature, report successful operations of both Ni- and Co- based catalysts (Guerrero-Ruiz et al., 1994; Matos, 2011; Xu et al., 2014b; Izhab et al., 2017; Zhang et al., 2017). Nonetheless, most of the reported experiences focus on AC or pyrolytic char selected or synthetized exclusively as catalytic supports. Conversely, in the present study char collected from an operating commercial gasifier, is considered. Unlike other carbonaceous supports, this material is generated as a by-product of the process and its properties were not accurately tuned for further catalytic applications. Therefore, at first, it was necessary to subject char to an extensive physicochemical characterization. Subsequently, char was treated for being used as support for Co-based catalyst synthesis. The synthesized catalysts were characterized as well, and tested for their DRM activity under identical operating conditions. Effects of metal loading, acid treatment of char with HNO_3_ and addition of MgO as promoter were investigated. Acid treatment results in the leaching of surface contaminants and inorganic compounds that could affect the reaction. Moreover, it is reported to enhance the methane cracking (R.5) increasing the H_2_ yield (Xu et al., 2014a). MgO was selected as promoter due to its capacity to hinder catalyst deactivation changing the nature of CO_2_ and CO chemisorbed species and thus inhibiting carbon deposition by the Boudouard reaction (R.6) on the working catalyst (Guerrero-Ruiz et al., 1993, 1994).

This study makes an attempt to valorize gasification char as catalyst support, at the laboratory scale. Furthermore, investigating and testing chars from commercial biomass gasifiers currently in operation, provides a better overview on the impacts of this specific application from an industrial perspective. Costs quantification and further conclusions on larger scales, could be drawn considering the scale-up of the process.

## Materials and Methods

### Catalyst Support

The catalyst supports are composed of char residues collected from one of the 46 small-scale biomass gasifiers operating in South Tyrol, Italy. The selected gasifier is based on a dual-stage technology (Wang et al., 2015; de Sales et al., 2017) and is designed to operate at 850°C and atmospheric pressure, using woodchips as feedstock and air as gasifying agent. This gasifier is characterized by a nominal thermal and electrical power output of 540 and 280 kW, respectively, from a throughput of about 230 kg/h of dry biomass.

The gasifier is designed such that, following the drying stage, the feedstock enters the pyrolysis reactor (200–700°C) where it is converted into tar-rich pyrolysis gases and char. Subsequently, the pyrolysis products are fed into the primary reactor, which is of floating fixed-bed type. In this reactor, the pyrolysis products are converted into higher-quality gas. Char and ashes are extracted from the bottom of the reactor. Thereafter, the char is humidified to decrease its losses by dispersion in air, and then collected in bags after the filtration system.

Char was withdrawn from the plant and taken to the laboratory for analysis. Firstly, it was washed with boiling deionized water and oven-dried at 105°C for 24 h, and secondly, it was characterized for its physico-chemical properties. Additionally, a fraction of the char acquired from the gasifier was heated in 0.1 M HNO_3_ solution at 105°C overnight to reduce the ash content and remove the surface contaminants, and again washed with deionized water until neutral pH was reached. The treated char was oven-dried at 105°C for 24 h. Even when more traditional AC is used as catalyst support, a washing step is always needed (Serp and Figueiredo, 2009). Therefore, possible costs arising from char washing will be very similar to the ones arising from more common AC washing.

### Catalyst Synthesis

The char supported cobalt catalysts were synthesized using wetness impregnation method. In order to investigate the effects of the metal loading on the catalyst performances, three catalysts with cobalt loading of 10, 15, and 20 wt.% were synthesized using untreated char as support, which were then referred to as Co10, Co15, and Co20.

Based on the performances of synthesized catalysts as a function of the metal loading, a metal loading of 10% was selected for the preparation of the other two catalysts, one supported on HNO_3_-treated char and one promoted by MgO (2%). They were then referred to as Co10/HNO_3_ and Co10/MgO. MgO was selected for its capacity of hindering carbon deposition, the main reason for DRM catalyst deactivation. Indeed, the basic character of MgO, as other alkaline and alkaline earth metal oxides, strengthen the chemisorption of an acidic compound such as CO_2_, favoring the reverse Bouduard reaction (R.6) and consequently the gasification of the deposited carbon (Guerrero-Ruiz et al., 1993, 1994).

The supports were mixed with an aqueous solution of cobalt nitrate hexahydrate (Co(NO_3_)_2_ · 6H_2_O) to obtain the desired metal loading. The solution with the support was stirred at 150°C until the complete evaporation of the solvent. The slurry was calcined in a muffle furnace at 400°C for 4 h. In the case of Co10/MgO, the impregnating mixture consisted of the aqueous solution of (Co(NO_3_)_2_ · 6H_2_O) and magnesium nitrate (Mg(NO_3_)_2_).

### Characterization

The elemental composition of char was determined using a Vario MACRO cube (Elementar) elemental analyzer, yielding its carbon, hydrogen, nitrogen and sulfur content. The ash content was measured according to UNI EN 14775:2010.

The thermal degradation of char, HNO_3_-treated char and all the synthesized catalysts was characterized in a simultaneous thermogravimetric analyzer (STA 449 F3, Netzsch) using both nitrogen and air as purging gases (20 mL min^−1^). Approximately, 10 mg of sample was heated from ambient temperature up to 1,000°C at a constant heating rate of 10°C min^−1^.

A 3Flex Surface Characterization Analyser (Micromeritics Co.) operating with N_2_ at −196.15°C was used for the determination of the surface area, pore volume and pore size of the samples. Before analysis, samples were dried for 24 h and vacuum degassed at 300°C for 3 h. The Brunauer-Emmett-Teller (BET) method (Brunauer et al., 1938) and the Barret-Joyner-Halenda (BJH) desorption analysis (Barrett et al., 1951), were used for the calculation of the specific area and the pore size distribution, respectively.

The structural features of the supports and the catalysts were investigated by X-ray diffraction (XRD) technique. X-ray powder diffraction patterns were collected at room temperature using a Philips X'Pert powder diffractometer (Bragg-Brentano parafocusing geometry) equipped with a focusing graphite monochromator on the diffracted beam and a proportional counter with electronic pulse height discrimination. A divergence slit of 0.5°, a receiving slit of 0.2 mm, an antiscatter slit of 0.5° were used, employing a nickel-filtered Cu Kα1 radiation (λ = 0.15406 nm) and a step-by-step technique (step of 0.05° for 20 s) with collection times of 10 s/step. Line broadening analysis was performed for the determination of the volume-weighted average crystallite size.

The morphological structure of the samples was investigated using a JEOL JEM 3010 transmission electron microscope (TEM) operating at 300 kV. The powder specimens were suspended in isopropyl alcohol and an aliquot of 5 μL was deposited on a copper grid (300 mesh) coated with carbon film. The copper grids were allowed to dry in air. The energy dispersive X-ray spectroscopy (EDX) measurements were carried out with an Oxford Instruments Isis System Series 300.

Temperature programmed reductions (TPR) were carried out in a lab-made equipment (Nichele et al., 2014). Samples (50 mg) were heated with a temperature rate of 10°C min^−1^ from 25°C to 800°C in a 5% H_2_/Ar flow (40 mL min^−1^) and the effluent gases were analyzed by a thermal conductivity detector (TCD). A moisture trap (magnesium perchlorate dihydrate) was located at the outlet to block the water which could be synthesized during reduction.

### Catalytic Tests

In each test, 0.2 g of dried char was loaded in a tubular quartz reactor 600 mm long, with an internal diameter of 8 mm. This corresponded to a bed height of ~18 mm. The char supported catalyst was itself sandwiched between a quartz wool bed. The furnace, along with the tubular reactor, was insulated with glass wool, to reduce the heat loss and maintain adiabatic conditions. Prior to each test, the samples were reduced in a pure hydrogen flow of 50 mL min^−1^, at 500°C with a ramp rate of 10°C min^−1^, for 12 h. After the reduction process, the catalytic reactor was cooled down in flowing hydrogen and then ramped at 10°C min^−1^ to the reaction temperature of 850°C in flowing nitrogen.

After reaching the operating temperature the N_2_ flow was stopped, and a gas mixture comprising of 49 vol.% CH_4_, 49 vol.% CO_2_ and 2 vol.% Ar was fluxed into the system by a mass flow controller (GasMix Zephyr, AlyTech) maintaining a weighted hourly space velocity (WHSV) of 6,500 mL g^−1^ h^−1^. The reaction temperature was held for the whole duration of the test (four hours), then the system was cooled down in pure N_2_ flow.

The product gasses from the reactor were analyzed using an on-line gas chromatograph (3000 micro-GC, SRA Instruments) equipped with two columns, a Molsieve column able to detect H_2_, O_2_, N_2_, CH_4_, and CO and a Plot-U column able to detect CO_2_, C_2_H_4_, C_2_H_6_, and C_3_H_6_/C_3_H_8_. The GC unit itself has a sampling valve, sampling 1 μl of the incoming gas every 3 min and circumventing the remaining gasses into the exhaust line.

**Figure 1:**
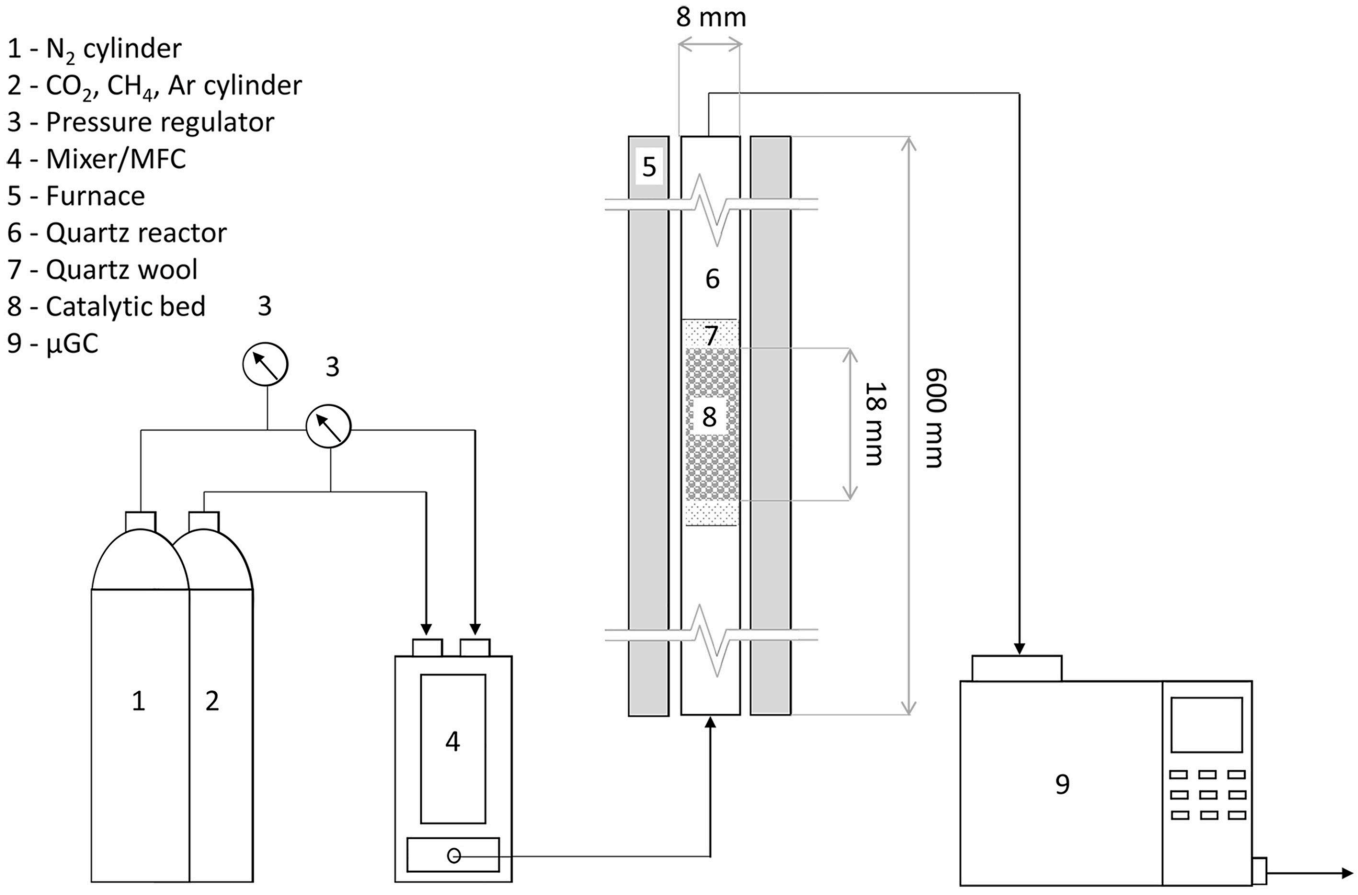
Lay-out of the experimental set-up for DRM.

Figure 1 shows the schematic of the experimental layout.

The catalytic performances of the samples were evaluated calculating the CO_2_ and CH_4_ conversions and H_2_ and CO yields according to the following equations (Song et al., 2008; Ayodele et al., 2016):

(Eq. 1) $$\begin{array}{r} CO_{2}\;conversion=\frac{ṅ\;CO_{2,in}-ṅ\;CO_{2,out}}{ṅ\;CO_{2,in}} \end{array}$$

(Eq. 2) $$\begin{array}{r} CH_{4}\;conversion=\frac{ṅ\;CH_{4,in}-ṅ\;CH_{4,out}}{ṅ\;CH_{4,in}} \end{array}$$

(Eq. 3) $$\begin{array}{r} H_{2}\;yield=\frac{ṅ\;H_{2,out}}{2\cdot ṅ\;CH_{4,in}} \end{array}$$

(Eq. 4) $$\begin{array}{r} CO\;yield=\frac{ṅ\;CO_{out}}{ṅ\;CH_{4,in}+ṅ\;CO_{2,in}} \end{array}$$

where ṅ *X* is the molar flow rate of the X specie, entering (in) or exiting (out) the reactor.

For comparison, average values measured after reaching stability were considered.

## Results and Discussion

### Characterization Results

#### Elemental Analysis

The elemental composition in terms of C, H, N, O, S, and the ash content of untreated and acid treated char are reported in Table 1. The results obtained for a commercial AC designed for catalytic applications (Norit GSX) are reported for comparison.

**Table 1:** Elemental analysis results and ash content of untreated char, char treated with HNO_3_ and a commercial activated carbon (AC).

	**Untreated char**	**HNO_**3**_-treated char**	**AC**
C % wt_dry_	91.4	76.8	90.6
H % wt_dry_	0.7	0.9	0.4
N % wt_dry_	0.3	1.3	0.5
S % wt_dry_	0.6	0.8	0.3
O^*^ % wt_dry_	2.9	18.2	3.8
Ash % wt_dry_	4.2	2.1	4.5

* *Calculated by difference*.

The results support the existence of similarities between char and AC. Both have a high carbon content, 91.4 and 90.6% respectively. In both cases, the ash amount is very low in comparison with other gasification char, 4.2% for char and 4.5% for AC. An ash content of 2.1% was measured for treated char proving the effectiveness of acid washing intended to reduce the presence of inorganic metals in the char matrix. Moreover, HNO_3_ treatment increased the N and O content from 0.3 to 1.3% and from 2.9 to 18.2%, respectively, while decreasing the C content from 91.4 to 76.8%. Indeed, through the acid washing, new oxygen-containing functional groups are created on the char surface. These oxygen groups could serve as chemically active anchoring sites for the metal particles leading to a good metal dispersion (Lahti et al., 2017).

#### Thermogravimetric Analysis

**Figure 2:**
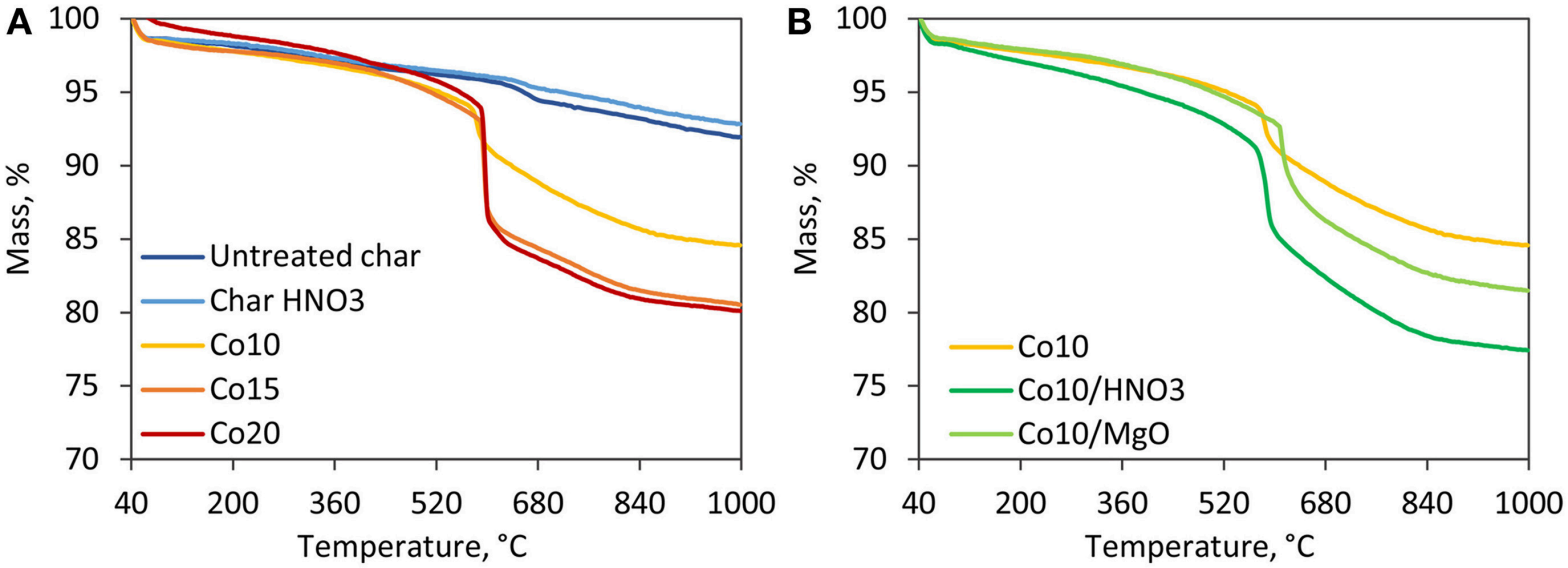
TGA results in inert atmosphere. **(A)** Comparison among supports and catalysts supported on untreated char with different Co loadings, **(B)** effect of acid washing and MgO promotion on catalysts with the same metal loading of 10%.

The results of thermal degradation in inert atmosphere are shown in Figure 2. For all char samples, the first degradation step observed in the temperature range of 40–100°C is associated to the loss of physisorbed water and the evaporation of the residual moisture (Bansal and Meenakshi, 2005). Untreated and acid-treated char behave similarly displaying a continuous mass loss due to the interaction between the freed surface oxygen groups and the carbon matrix (Bansal and Meenakshi, 2005). All the five catalysts show a thermal degradation behavior different from the supports registering a sharp mass loss around 600°C. This is associated to the combined effect of cobalt oxides reduction and loss of non-graphitic carbon. It can be seen from Figure 2A, that higher metal loadings, and therefore higher content of oxides, are associated to higher mass losses. While this temperature zone is crucial for DRM reactions, the catalysts are reduced in H_2_ prior to the activity tests. Since no cobalt oxide species are prevalent on the catalyst surface, post-reduction, negligible mass loss is expected under DRM conditions. Figure 2B shows the effects of acid washing and MgO promotion. Thermal degradation is favored by HNO_3_ treatment. On the other hand, the presence of MgO shifted the highest degradation rates to slightly higher temperatures favoring the thermal stability of the catalyst.

**Figure 3:**
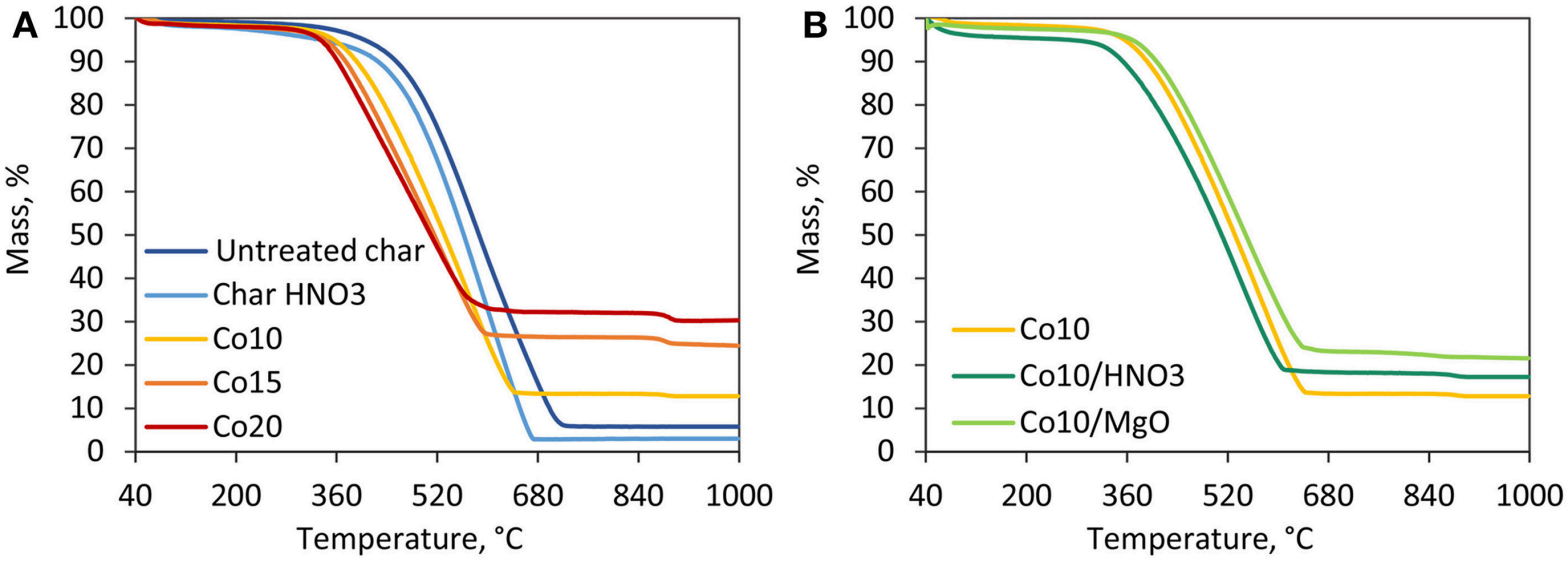
TGA results in oxidative atmosphere. **(A)** Comparison among supports and catalysts supported on untretaed char with different Co loadings, **(B)** effect of acid washing and MgO promotion on catalysts with the same metal loading of 10%.

Figure 3 shows the results obtained under an oxidative atmosphere. Untreated and treated char show a similar trend, as observed earlier. Treated char is more likely to degrade at lower temperature than untreated char. The residual mass associated to the presence of inorganic compounds in this case is lower than untreated char proving the effectiveness of the acid washing. Catalysts are more reactive than the supports (Davis and Occelli, 2016). Indeed, the introduction of cobalt oxides favor the degradation of the material. The maximum mass loss of 70–85 wt.% occurs in the temperature range of 350–640°C. Beyond this temperature, a mass loss of ~2 wt.% is displayed around 900°C, associated to the transition of Co_3_O_4_ to more stable CoO (Wigzell and Jackson, 2017). Figure 3A shows the effect of the metal loading where higher cobalt loadings are associated to lower degradation temperatures and of course to higher residual masses (i.e., ashes and metallic cobalt). Figure 3B shows that HNO_3_ promoted thermal degradation of the catalyst, while MgO increased its thermal stability.

#### Physisorption Analysis

**Figure 4:**
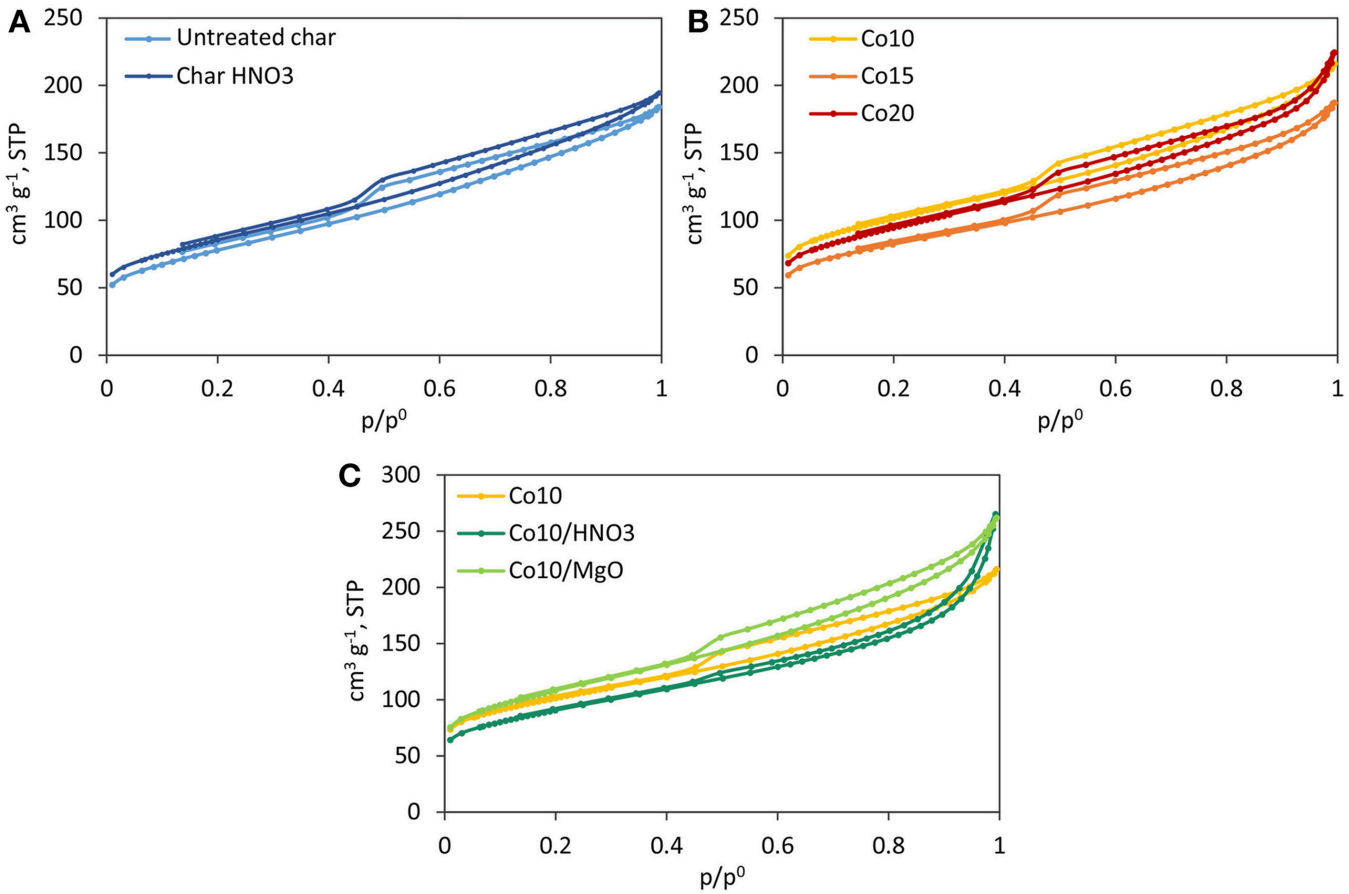
Adsorption isotherms (N_2_, −196°C). **(A)** Supports, **(B)** effect of metal loading, **(C)** effect of HNO_3_ treatment of char and MgO promotion.

All catalysts and supports show similar adsorption-desorption isotherms shapes (Figure 4). According to the BDDT (Brunnauer-Deming-Deming-Teller) classification (Brunauer et al., 1940), they can be classified as type IV isotherms, typical of mesoporous structures. At relative pressures higher than 0.45, they display type III and IV hysteresis loops indicative of the presence of slit pores. Only isotherms corresponding to Co20 and Co10/MgO, take on a hyperbolic shape close to p/p_0_ = 1. This is due to a greater presence of macropores and it is confirmed by the higher pore size associated (5.4 and 6.9 nm) (Gregg and Sing, 1982).

Physisorption results for untreated and treated char presented in Table 2 are very similar, indicating that char did not undergo any relevant structural change during acid washing. They both have S_BET_ values (297 and 295 m^2^ g^−1^ for untreated and treated char, respectively) that are above the average values reported in the literature for other gasification chars (Benedetti et al., 2018).

**Table 2:** Physisorption analysis results.

	**Supports**		**Catalysts**					
	**Untreated char**	**HNO_**3**_ char**	**Co10**	**Co15**	**Co20**	**Co10/HNO_**3**_**	**Co10/MgO**	**Char treated as catalyst**
S_BET_ m^2^ g^−1^	297	295	364	294	337	323	386	642
Pore volume cm^3^ g^−1^	0.26	0.25	0.26	0.23	0.28	0.35	0.33	0.44
Pore size nm	4.5	4.6	4.9	5.1	5.4	6.9	5.3	5.2

Usually, during catalyst synthesis, the added metals block the pores leading to a decrease in the surface area and pore volume of the material (Ail et al., 2018). Nevertheless, such a phenomena was not observed for char supported catalysts (see Table 2). In this case, S_BET_, pore volume and pore size increased (except for Co15). This can be attributed to the modification of the char structure during catalyst synthesis. In fact, when char undergoes the same treatment as for catalyst preparation (without metal precursor), due to the combined effect of intense mixing, washing and calcination in air, volatiles leave the carbon matrix of char widening its porous structure, and thus, increasing both its surface area and pore volume. According to Table 2, S_BET_ increased from 297 to 642 m^2^ g^−1^ while pore volume from 0.26 to 0.44 cm^3^ g^−1^. Both parameters have almost doubled. Referring to these values as starting points, as expected, a decrease in all the parameters was registered after catalyst preparation.

#### XRD

**Figure 5:**
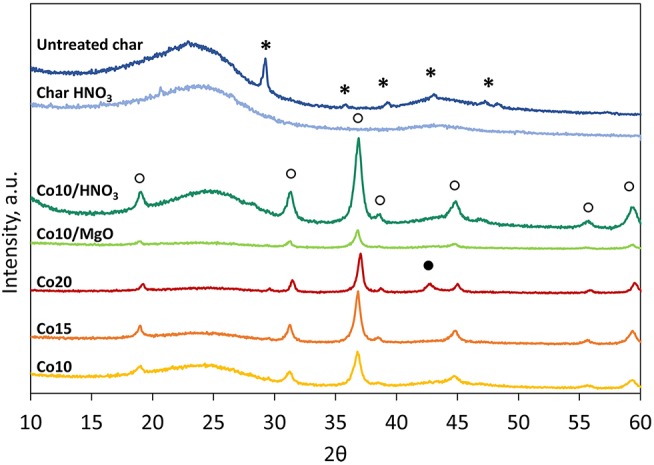
XRD patterns of supports and calcinated catalysts (^*^, CaCO_3_; ◦, Co_3_O_4_; •, CoO).

Figure 5 illustrates the XRD patterns of the supports and the catalysts. In general two phases can be distinguished: an amorphous phase related to the carbon structure and a crystalline phase related to the presence of crystalline compounds.

Untreated char shows only peaks attributed to calcite (CaCO_3_). Compared with other chars derived from commercial biomass gasifiers, the selected char contains less crystalline compounds and therefore it is more suitable for catalytic applications than others. The effectiveness of the acid washing is additionally highlighted by the XRD spectrum of the treated char that does not display any peak but pursues a typical spectrum of pure carbonaceous materials.

Patterns associated to the five catalysts show peaks related to the presence cobalt (II, III) oxide (Co_3_O_4_). Only Co20 catalyst shows also peaks corresponding to cobalt (II) oxide (CoO). MgO peaks (36.9°and 42.9°, JCPDS 87-0653) were not clearly detectable in Figure 5 in the case of MgO promoted catalysts due to their partial overlapping with the Co_3_O_4_ peaks (36.8° and 44.8°, JCPDS 74-1656) (Mirzaei et al., 2015).

**Figure 6:**
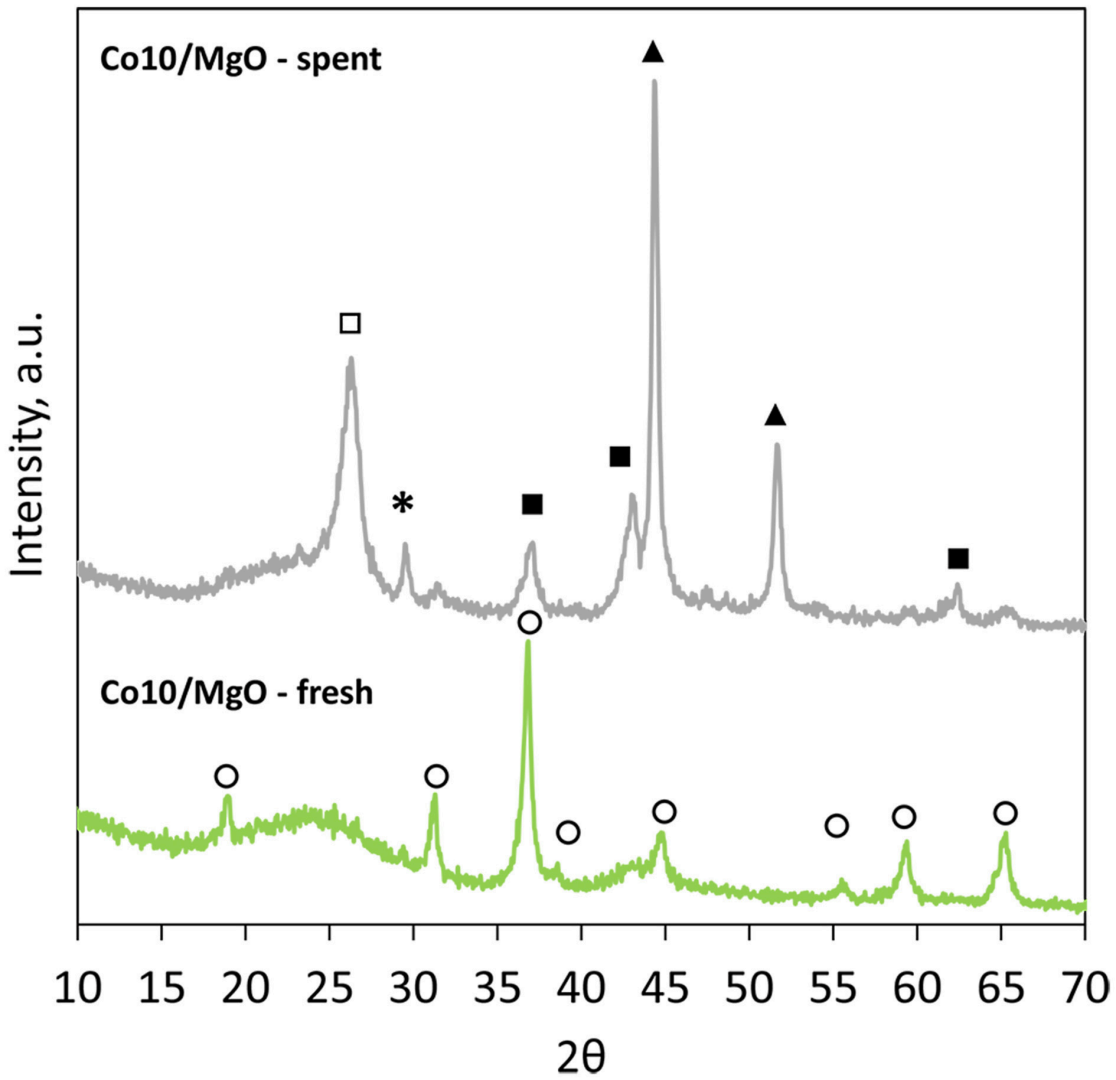
XRD patterns of fresh and spent Co10/MgO (^*^, CaCO_3_; ◦, Co_3_O_4_; ■, MgO; ▴, Co; □, graphite).

However, Figure 6, that reports the patterns of fresh and spent Co10/MgO, highlights the presence of a small broad peak of MgO at 42.9° that could be correlated to a population of very small particles with an average size smaller than 3 nm (Riello et al., 1998).

Also the XRD pattern of spent Co10/MgO reveals the presence of MgO. Other peaks are related to metallic cobalt, calcite and graphite (002). There is general agreement that the catalytic deactivation during the CO_2_ reforming of methane is caused by two different deactivation mechanisms, namely, oxidation of metallic sites, and carbon deposition (Ruckenstein and Wang, 2000). The presence of metallic cobalt in the spent catalyst indicates that no oxidation of the metal took place. On the other hand, the presence of graphitic carbon, less reactive to CO_2_ than the amorphous carbon of the support (Xu et al., 2014a), is indicative of carbon deposition. However, the good stability of Co10/MgO during the test proved that in this case, carbon deposition was not detrimental for the process. The average crystallite sizes are reported in Table 3. Metal loading affects particle size. Higher metal loadings are associated to bigger particles. Co10/HNO_3_ shows an increased particle size compared to Co10. It is important to point out that the calculation of the Co_3_O_4_ particle sizes reported for Co10/MgO, could be distorted by the presence of underlying MgO peaks.

**Table 3:** Cobalt oxides particle sizes calculated from the XRD results.

	**Phase**	**Particle size, nm**
Co10	Co_3_O_4_	9
Co15	Co_3_O_4_	13
Co20	Co_3_O_4_	19
	CoO	12
Co10/HNO_3_	Co_3_O_4_	13
Co10/MgO	Co_3_O_4_	16

#### TEM

Due to its peculiar crystalline structure and remarkable performances (as it will be discussed in the DRM results section), sample Co10/MgO has been further analyzed by TEM.

**Figure 7:**
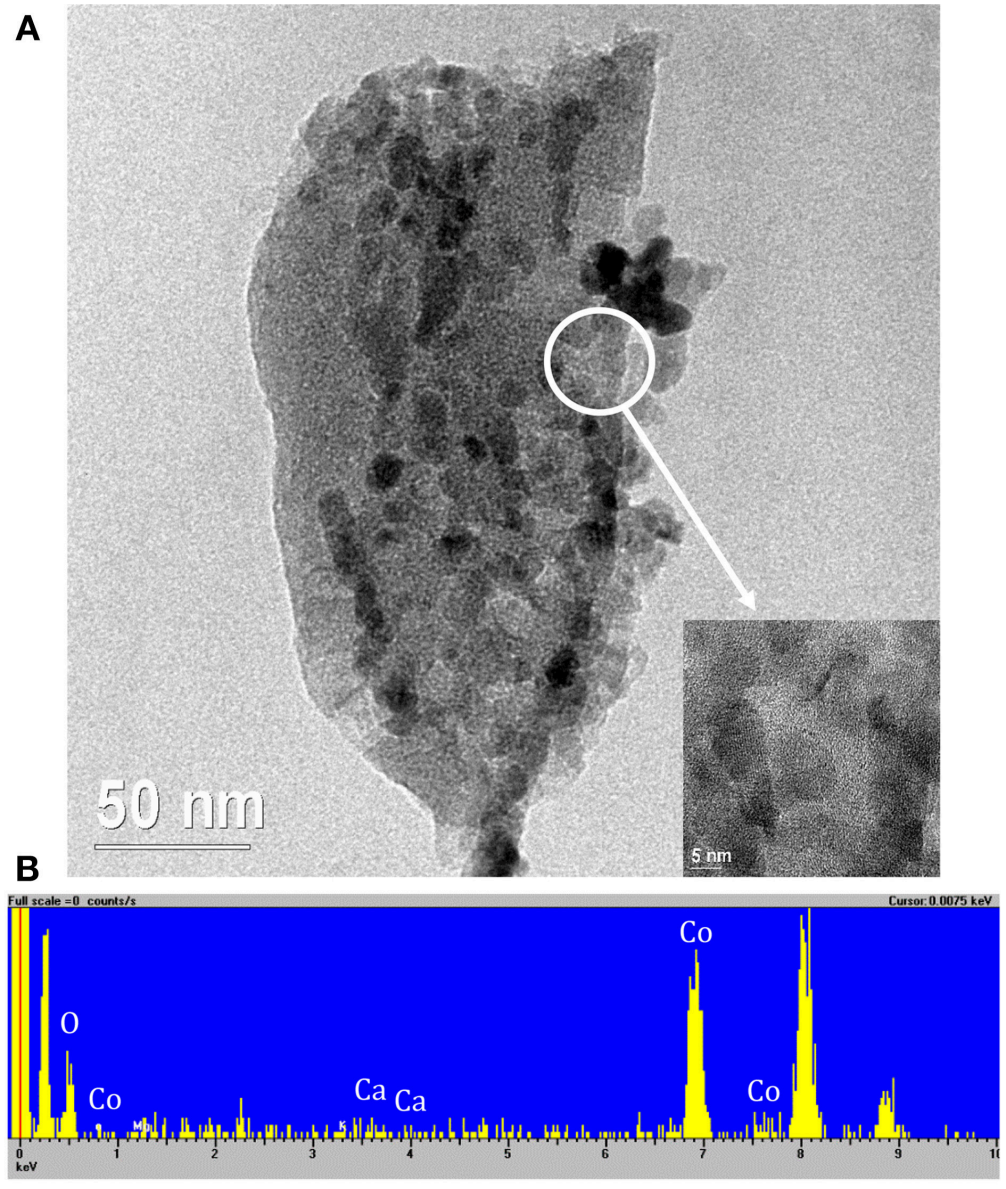
**(A)** Representative TEM micrographs of Co10/MgO. The inset reports the magnification of the area delimited by the white circle, **(B)** EDX spectrum. The peaks with no label correspond to the presence of C and Cu of the instrument grid.

Figure 7A shows the presence of particles dispersed on the char of about 15 nm supporting the XRD findings. The inset of Figure 7A refers to the magnification of the zone delimited by the white circle. It displays the presence of crystal planes into the particles that, according to the EDX analysis (Figure 7B), are made of Co. No traces of Mg were detected.

**Figure 8:**
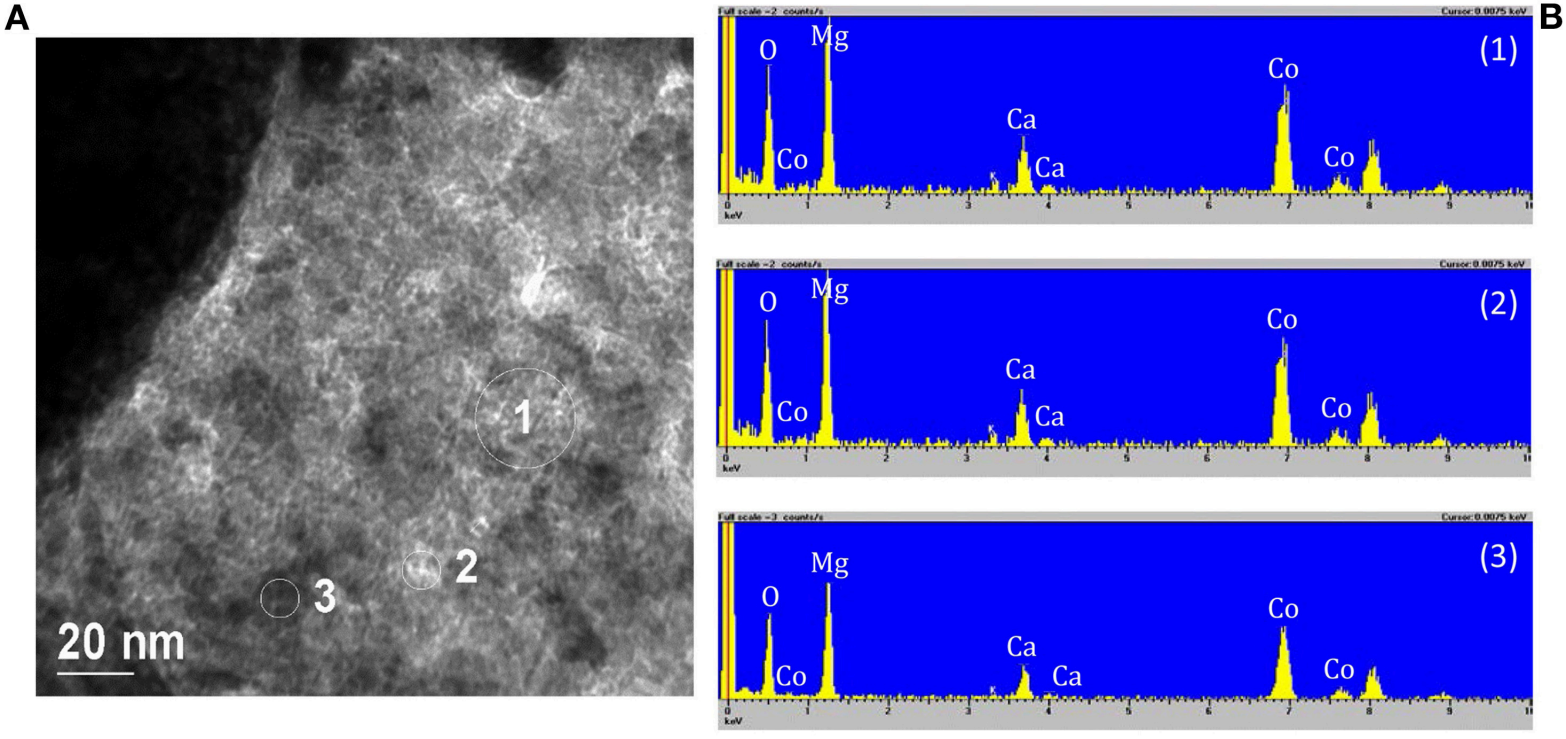
**(A)** Representative TEM micrographs of the sample Co10/MgO. Area 1, 2, and 3 were analyzed by EDX, **(B)** EDX spectra relative to the circled zones. The peaks with no label correspond to the presence of C and Cu of the instrument grid.

In order to highlight the presence of MgO, it was necessary to analyze several parts of the samples by EDX. In fact, the sample microstructure is very irregular and complex, the size of MgO particles is of about 1 nm or less, and the Mg atom is very light so that electronic contrast is very low. Numbers 1, 2, and 3 in Figure 8A correspond to the circled zone analyzed by EDX and showed in Figure 8B. The white and very small “particles” are optical artifacts that disappear at higher magnifications. All the zones investigated gave the same results: there is a coexistence of the Mg and Co signal that does not correspond to the presence of well distinct particles. Therefore, it was not possible to isolate the signal of the single MgO particles.

#### TPR

**Figure 9:**
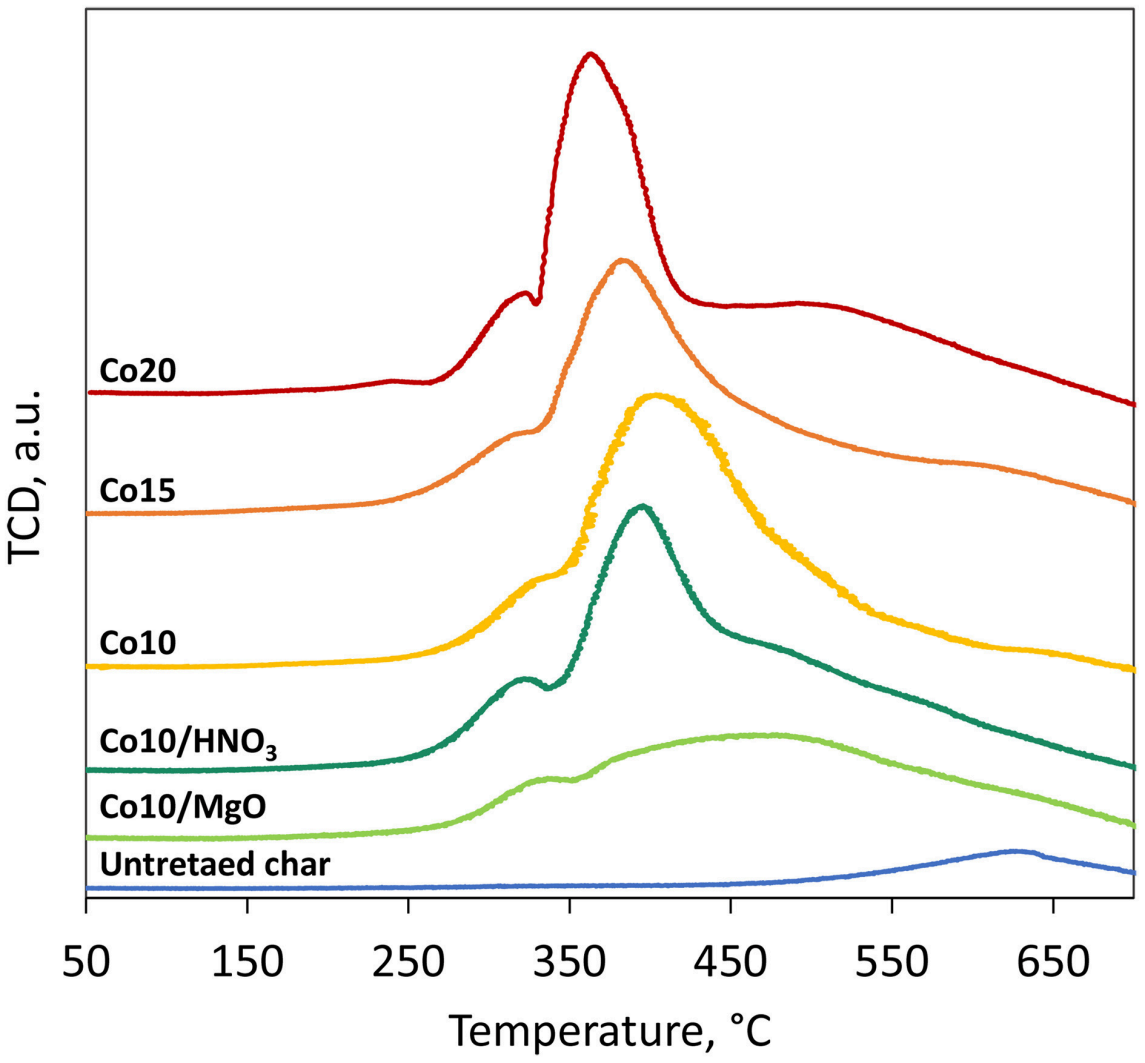
TPR results.

Results of the temperature-programmed reduction (TPR) are illustrated in Figure 9. Three main peaks are shown for all the five catalysts. The first one is associated to the reduction of Co_3_O_4_ to CoO and the second one to the reduction of CoO to metallic cobalt Co^0^ (Ail et al., 2018). The H_2_ consumption peak observed at T > 550°C for all the samples, untreated char included, is attributed to the interaction of H_2_ with the surface oxygen containing groups on the char surface. No peaks are displayed at T > 650°C leading to the conclusion that no metal-support compounds were formed during catalysts synthesis.

At higher metal loadings, the reduction peaks are narrower and shifted to lower temperature indicating the presence of larger particles (as confirmed by XRD results) that are easier to be reduced (Trépanier et al., 2010). The effect of HNO_3_ treatment on the catalysts leads to narrower reduction peaks at lower reduction temperatures compared to the catalysts supported on untreated char, owing to the larger particle size of the active metal. MgO promoted catalyst shows wider peaks compared to other catalysts. The broad peak could be associated to the simultaneous reduction of Co_3_O_4_ and small fractions of MgO.

### DRM Tests Results

**Figure 10:**
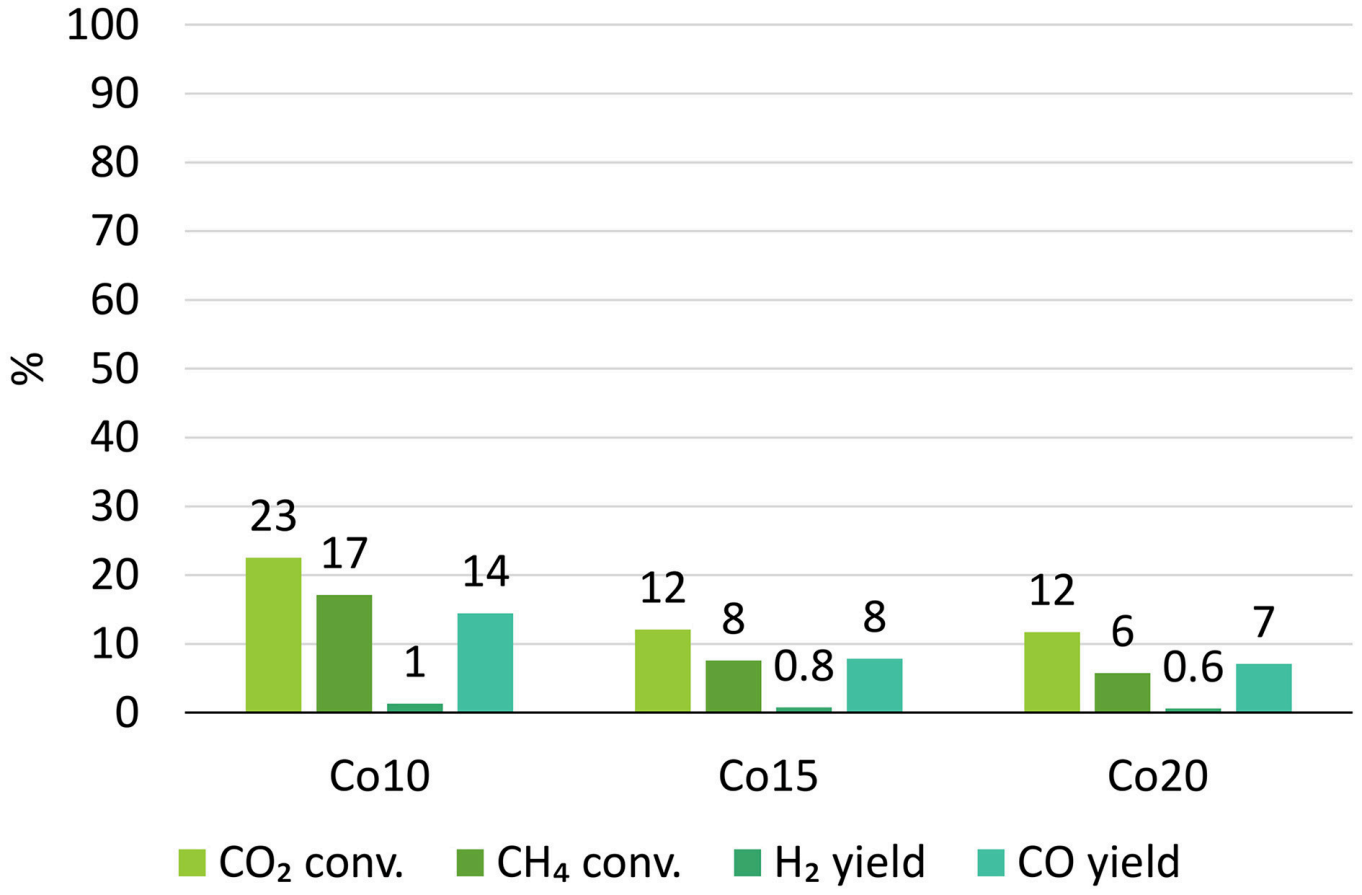
DRM results using untreated char as catalyst support—Effects of cobalt loading.

Figure 10 shows the results of the catalytic tests run with the three catalysts supported on untreated char. Average values measured after reaching stability were considered.

While the activity of the synthesized catalysts started with high initial conversions (e.g., 73% CO_2_ conversion and 46% CH_4_ conversion for Co10), the catalysts deactivated after 1 hour.

Furthermore, the CH_4_ and CO_2_ conversions decreased with increasing cobalt loading. The average CO_2_ conversion decreased from 23 to 12% while the average CH_4_ conversion from 17 to 6%. Consequentially, the H_2_ yield dropped from 1 to 0.6% and the CO yield from 14 to 7%.

Our results are confirmed by a study of Budiman et al. who stated that a cobalt loading of 10% is the best compromise between low carbon deposition and low deactivation (Budiman et al., 2012). According to them, in DRM reactions with low cobalt loading the generation of oxygen species from CO_2_ is so fast that the cobalt active sites are more likely to be oxidized into inactive species than the carbon derived by the methane decomposition. On the contrary, high Co loadings lead to an excessive deposition of carbon by CH_4_ decomposition that CO_2_ activation cannot oxidize.

Moreover, high metal loading may be associated with an excessive quantity of cobalt that, incapable to penetrate the matrix of the catalyst, forms Co agglomerates on the surface blocking micropores and decreasing the portion of the surface available for the reaction (Zhang et al., 2014a).

Results indicated in Figure 10 show that CO_2_ conversions are always higher than CH_4_ conversions. This is due to the combined effect of the reverse Bouduard reaction (R.6) and reverse water gas shift reaction (R.4) that consume CO_2_ (Haghighi et al., 2007).

It is observed that, compared to the data reported in the literature for cobalt catalysts supported on AC, the calculated conversions and yields, in this work are considerably low. Zhang et al. (2014b) measured at 850°C CO_2_ and CH_4_ conversions of 72 and 65%, which are 3–4 times higher than what it is reported in this study, at much higher WHSV of 72,000 mL h^−1^ ${\text{g}}_{\text{cat}}^{-1}$. Guerrero-Ruiz et al. (1994) reported CO_2_ and CH_4_ conversions of 33.4 and 15.2% and H_2_ and CO yields of 9.3 and 25.9%. Although Izhab et al. (2017) measured lower CO_2_ (17%) and CH_4_ (14%) conversions than what reported in Figure 10, the H_2_ (40%) and CO (20%) yields were superior to our results.

The differences in the results could be associated with the origin of char and the degree of its treatment compared to the commercial activated carbon. Moreover, the differences in the methods of catalyst synthesis and reduction conditions play a significant role.

**Figure 11:**
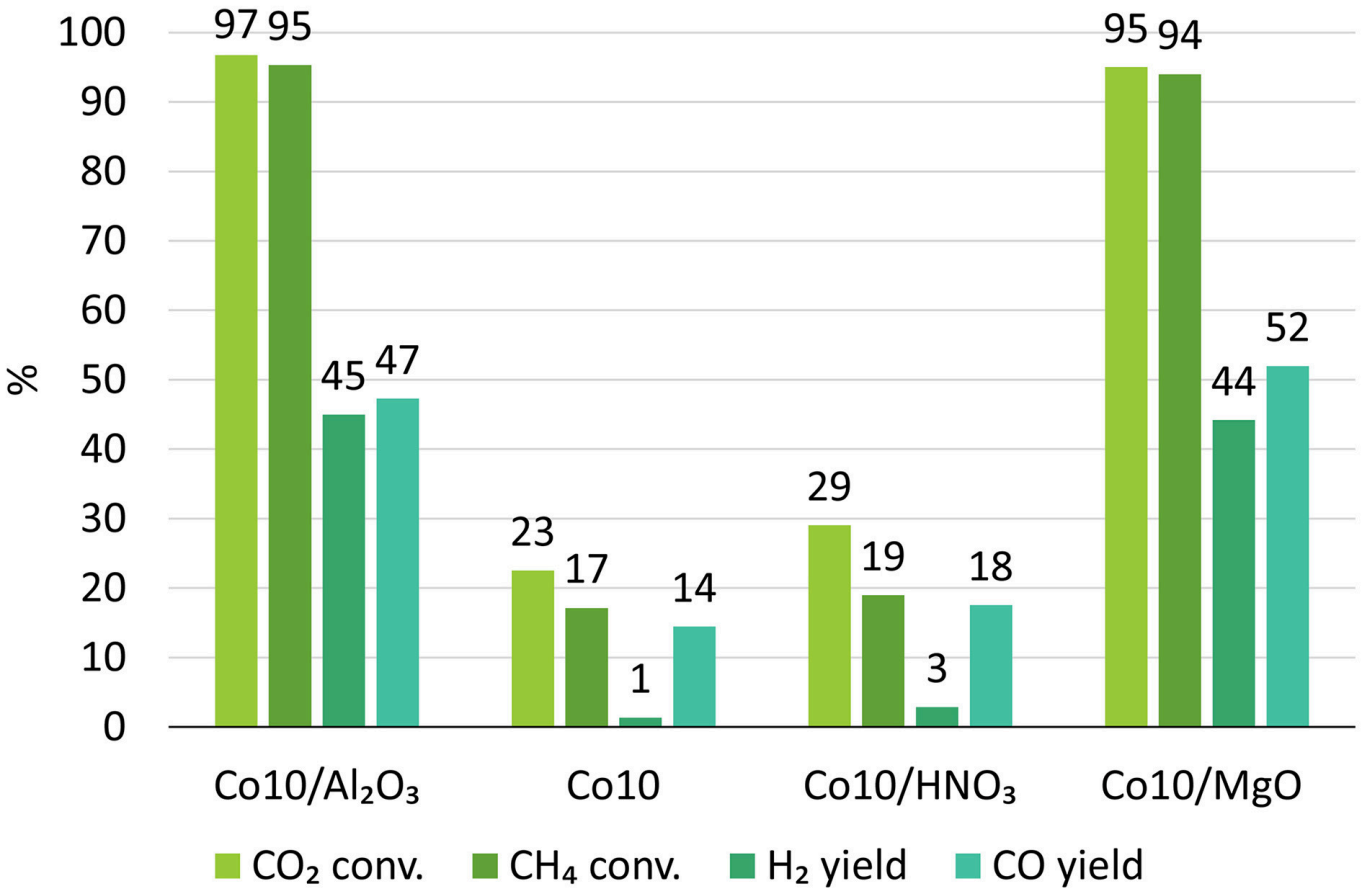
DRM results for catalyst supported on more traditional alumina (Budiman et al., 2012), on char treated with HNO_3_ and on untreated char with the addition of MgO.

The activity of char supported catalysts were also compared to Co-catalysts supported on alumina, which is a more conventional support for DRM reactions (Budiman et al., 2012). 10Co/Al_2_O_3_ catalysts resulted in a CO_2_ conversion of 97% and CH_4_ conversion of 95% with a yield of 45% H_2_ and 47% CO (Figure 11).

Clearly, gasification derived char cannot be used as support as it is. It demands additional treatments or alkali promotions to enhance its catalytic performances and increase its stability. For this reason, 10Co catalysts were synthesized using char treated with HNO_3_ and MgO as promoter. Results are shown in Figure 11.

HNO_3_ treatment resulted in slightly higher activities and yields and slower deactivation in respect to other catalysts. The higher catalyst activity might be due to the increased acidity of the support that may favor CH_4_ cracking with associated H_2_ production (R.5) (Xu et al., 2014a). However, according to R.5, coupled with an improved ability of extracting H_2_, there is also an increase in carbon generation that will deposit and eventually lead to the catalyst deactivation.

On the contrary, the presence of MgO drastically increased the conversions and the yields up to 95 and 94% for CO_2_ and CH_4_ conversions, and 44 and 53% for H_2_ and CO yields. Moreover, the catalyst proved to be stable during the whole duration of the test.

**Figure 12:**
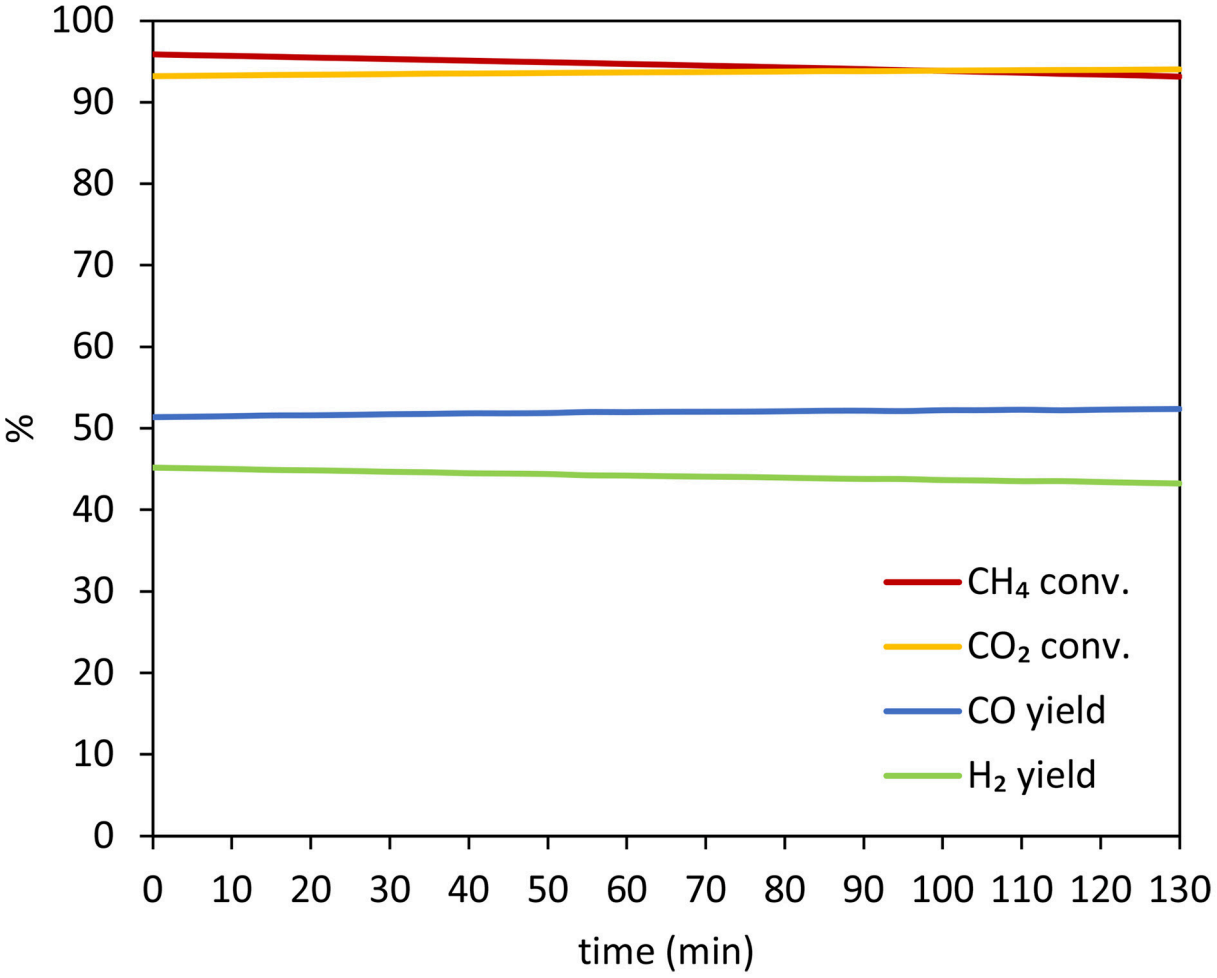
Variation of CO_2_ and CH_4_ conversion and H_2_ and CO yield with time for 10Co/MgO, after reaching stability.

In this regards, Figure 12 shows the variation of CO_2_ and CH_4_ conversion and H_2_ and CO yield with time for 10Co/MgO, after reaching stability.The reasons for the improved performance of 10Co/MgO are two-fold. Firstly, MgO and CoO have very close lattice parameters so they are completely miscible and can form solid solution with a good resistance to sintering phenomena (Budiman et al., 2012). Secondly, as a promoter, MgO migrates to the deactivating sites where methane cracking take place (R.5) and oxidizes itself the deposited carbon and/or catalyzes the reverse Bouduard reaction (R.6) (Budiman et al., 2012). In fact, through MgO addition, the concentration of Lewis basicity of the support is increased and thus the chemisorption of CO_2_, acid gas, is enhanced (Guerrero-Ruiz et al., 1993, 1994).

## Conclusion

In regard to the reuse of gasification-derived-char, its application in the synthesis of catalysts for dry reforming of methane exhibited promising results. DRM has been selected as test reaction due its potential to reduce CO_2_ emissions and effectively store in a chemical form the energy excess derived by renewable energy sources, usually fluctuant, and intermittent.

Since char itself could not be used directly in the catalyst synthesis process, it demanded vigorous treatments, such as leaching with nitric acid and even promotion with alkaline earth metal oxides like MgO, for its effective use.

Investigations on the optimum metal loading for synthesizing char supported cobalt catalysts revealed 10 wt.% as the ideal catalyst configuration. Additionally, HNO_3_ washing of char enhanced the catalyst activity by removing surface contaminants and also by amplified CH_4_ cracking favored by the increased acidity of the support. The DRM activity and syngas yields were improved significantly when the catalysts were promoted by 2 wt.% MgO, which alters the nature of CO_2_ and CO chemisorbed species, inhibiting carbon deposition on the catalyst surface by the Boudouard reaction.

Therefore, with a catalyst activity comparable to conventional DRM, gasification char can be used as catalyst support. With appropriate treatment techniques and structural promotions, the utilization of char supported catalysts can be extended to other reactions as well. Eventually, these carbonaceous residues could be thought as a valuable resource, and not as an industrial waste.

## Author Contributions

VB and SA synthetized, characterized, and tested the char-supported catalysts. VB and SA wrote the manuscript with assistance from FP and MB. FP and MB supervised the research.

### Conflict of Interest Statement

The authors declare that the research was conducted in the absence of any commercial or financial relationships that could be construed as a potential conflict of interest.

## References

[B1] Ahmad J. Rashid U. Patuzzi F. Baratieri M. Taufiq-Yap Y. H. (2018). Synthesis of char-based acidic catalyst for methanolysis of waste cooking oil: an insight into a possible valorization pathway for the solid by-product of gasification. Energy Convers. Manag. 158, 186–192. 10.1016/j.enconman.2017.12.059

[B2] Ail S. S. Benedetti V. Baratieri M. Dasappa S. (2018). Fuel-rich combustion synthesized Co/Al2O3 catalysts for wax and liquid fuel production via Fischer-Tropsch reaction. Ind. Eng. Chem. Res. 57, 3833–3843. 10.1021/acs.iecr.7b04174

[B3] Ail S. S. Dasappa S. (2016). Investigations into enhanced wax production with combustion synthesized Fischer–Tropsch catalysts. Energy Convers. Manag. 116, 80–90. 10.1016/j.enconman.2016.02.075

[B4] Arora S. Prasad R. (2016). An overview on dry reforming of methane: strategies to reduce carbonaceous deactivation of catalysts. RSC Adv. 6, 108668–108688. 10.1039/C6RA20450C

[B5] Ayodele B. V. Khan M. R. Cheng C. K. (2016). Production of CO-rich hydrogen gas from methane dry reforming over Co/CeO_2_ catalyst. Bull. Chem. React. Eng. Catal. 11:210 10.9767/bcrec.11.2.552.210-219

[B6] Bansal R. C. Meenakshi G. (2005). Activated Carbon Adsorption. Boca Raton, FL: Group, Taylor and Francis 10.1201/9781420028812

[B7] Barrett E. P. Joyner L. G. Halenda P. P. (1951). The determination of pore volume and area distributions in porous substances I. Computations from nitrogen isotherms. J. Am. Chem. Soc. 73, 373–380. 10.1021/ja01145a126

[B8] Basso D. Patuzzi F. Antolini D. Ail S. S. Cordioli E. Benedetti V. (2018). Novel extension of biomass poly-generation to small scale gasification systems in South-Tyrol in 26th European Biomass Conference and Exhibition (Copenhagen: ETA-Florence Renewable Energies).

[B9] Basu P. (2010). Biomass Gasification and Pyrolysis Practical Design and Theory. Oxford: Elsevier Academic Press.

[B10] Benedetti V. Patuzzi F. Baratieri M. (2018). Characterization of char from biomass gasification and its similarities with activated carbon in adsorption applications. Appl. Energy 227, 92–99. 10.1016/j.apenergy.2017.08.076

[B11] Brunauer S. Deming L. S. Edwards Deming W. Teller E. (1940). On a theory of the van der Waals adsorption of gases. J. Am. Chem. Soc. 7, 1723–1732. 10.1021/ja01864a025

[B12] Brunauer S. Emmett P. H. Teller E. (1938). Gases in multimolecular layers. J. Am. Chem. Soc. 60, 309–319. 10.1021/ja01269a023

[B13] Budiman A. W. Song S.-H. Chang T.-S. Shin C.-H. Choi M.-J. (2012). Dry reforming of methane over oobalt catalysts: a literature review of catalyst development. Catal. Surv. Asia 16, 183–197. 10.1007/s10563-012-9143-2

[B14] Chattanathan S. A. Adhikari S. McVey M. Fasina O. (2014). Hydrogen production from biogas reforming and the effect of H_2_S on CH_4_ conversion. Int. J. Hydrogen Energy 39, 19905–19911. 10.1016/j.ijhydene.2014.09.162

[B15] Chen X. Wang F. Yan X. Cheng Z. Han Y. Jie Z. (2018). Thermal and chemical analysis of methane dry reforming in a volumetric reactor under highly concentrated solar radiation. Sol. Energy 162, 187–195. 10.1016/j.solener.2018.01.032

[B16] Davis B. H. Occelli M. L. (2016). Fischer-Tropsch Synthesis, Catalysts, and Catalysis: Advances and Applications. Boca Raton, FL: CRC Press 10.1201/b19455

[B17] de Sales C. A. V. B. Maya D. M. Y. Lora E. E. S. Jaén R. L. Reyes A. M. M. González A. M. (2017). Experimental study on biomass (*Eucalyptus* spp.) gasification in a two-stage downdraft reactor by using mixtures of air, saturated steam and oxygen as gasifying agents. Energy Convers. Manag. 145, 314–323. 10.1016/j.enconman.2017.04.101

[B18] Edwards J. H. Maitra A. M. (1995). The chemistry of methane reforming with carbon dioxide and its current and potential applications. Fuel Process. Technol. 42, 269–289. 10.1016/0378-3820(94)00105-3

[B19] Galhetas M. Lopes H. Freire M. Abelha P. Pinto F. Gulyurtlu I. (2012). Characterization, leachability and valorization through combustion of residual chars from gasification of coals with pine. Waste Manag. 32, 769–779. 10.1016/j.wasman.2011.08.021 21963044

[B20] Gao J. Hou Z. Lou H. Zheng X. (2011). Dry (CO_2_) reforming. Fuel Cells 7, 191–221. 10.1016/B978-0-444-53563-4.10007-0

[B21] Gregg S. J. Sing K. S. W. (1982). Adsorption, Surface Area and Porosity. 2nd Edn. London: Academic Press.

[B22] Guerrero-Ruiz A. Rodrìguez-Ramos I. Sepùlveda-Escribano A. (1993). Effect of the basic function in Co, MgO/C catalysts on the selective oxidation of methane by carbon dioxide. J. Chem. Soc. Chem. Comm. 487–488. 10.1039/C39930000487

[B23] Guerrero-Ruiz A. Sepùlveda-Escribano A. Rodrìguez-Ramos I. (1994). Cooperative action of cobalt and MgO for the catalysed reforming of CH_4_ with CO_2_. Catal. Today 21, 545–550. 10.1016/0920-5861(94)80178-9

[B24] Haghighi M. Sun Z. Wu J. Bromly J. Ling H. Ng E. (2007). On the reaction mechanism of CO_2_ reforming of methane over a bed of coal char. Proc. Combust. Inst. 31, 1983–1990. 10.1016/j.proci.2006.07.029

[B25] Hansen V. Müller-Stöver D. Ahrenfeldt J. Holm J. K. Henriksen U. B. Hauggaard-Nielsen H. (2015). Gasification biochar as a valuable by-product for carbon sequestration and soil amendment. Biomass Bioenergy 72, 300–308. 10.1016/j.biombioe.2014.10.013

[B26] Izhab I. Aishah N. Amin S. Teknologi U. (2017). Dry reforming of methane over oil palm shell activated carbon and ZSM-5 supported cobalt catalysts. Int. J. Green Energy 14, 831–888. 10.1080/15435075.2017.1334659

[B27] Klinghoffer N. B. Castaldi M. J. Nzihou A. (2012). Catalyst properties and catalytic performance of char from biomass gasification. Ind. Eng. Chem. Res. 51, 13113–13122. 10.1021/ie3014082

[B28] Lahti R. Bergna D. Romar H. Hu T. Comazzi A. Pirola C. (2017). Characterization of cobalt catalysts on biomass-derived carbon supports. Top. Catal. 60, 1415–1428. 10.1007/s11244-017-0823-z

[B29] Lavoie J.-M. (2014). Review on dry reforming of methane, a potentially more environmentally-friendly approach to the increasing natural gas exploitation. Front. Chem. 2:81. 10.3389/fchem.2014.00081 25426488 PMC4227528

[B30] Marsh H. Rodriguez-Reinoso F. (2006). Activated Carbon. Amsterdam: Elsevier Science and Technology Books 10.1016/B978-008044463-5/50016-9

[B31] Matos J. (2011). Promoter effect upon activated carbon- supported Ni-based catalysts in dry methane reforming. Eurasian Chem. J. 14, 12–15. 10.18321/ectj91

[B32] Mendiara T. García-Labiano F. Abad A. Gayán P. de Diego L. F. Izquierdo M. T. (2018). Negative CO_2_ emissions through the use of biofuels in chemical looping technology: a review. Appl. Energy 232, 657–684. 10.1016/j.apenergy.2018.09.201

[B33] Mirzaei F. Rezaei M. Meshkani F. Fattah Z. (2015). Carbon dioxide reforming of methane for syngas production over Co – MgO mixed oxide nanocatalysts. J. Ind. Eng. Chem. 21, 662–667. 10.1016/j.jiec.2014.03.034

[B34] Muradov N. Smith F. T-Raissi A. (2005). Catalytic activity of carbons for methane decomposition reaction. Catal. Today 102–103, 225–233. 10.1016/j.cattod.2005.02.018

[B35] Nichele V. Signoretto M. Pinna F. Menegazzo F. Rossetti I. Cruciani G. (2014). Ni/ZrO_2_ catalysts in ethanol steam reforming: inhibition of coke formation by CaO-doping. Appl. Catal. B Environ. 150–151, 12–20. 10.1016/j.apcatb.2013.11.037

[B36] Nikoo M. K. Amin N. A. S. (2011). Thermodynamic analysis of carbon dioxide reforming of methane in view of solid carbon formation. Fuel Process. Technol. 92, 678–691. 10.1016/j.fuproc.2010.11.027

[B37] Patuzzi F. Prando D. Vakalis S. Rizzo A. M. Chiaramonti D. Tirler W. (2016). Small-scale biomass gasification CHP systems: comparative performance assessment and monitoring experiences in South Tyrol (Italy). Energy 112, 285–293. 10.1016/j.energy.2016.06.077

[B38] Riello P. Canton P. Benedetti A. (1998). Au/C catalyst: experimental evidence of the coexistence of nanoclusters and larger Au particles. Langmuir 14, 6617–6619. 10.1021/la9803272

[B39] Ruckenstein E. Wang H. Y. (2000). Carbon dioxide reforming of methane to synthesis gas over supported cobalt Catalysts 204, 257–263. 10.1016/S0926-860X(00)00674-8

[B40] Runtti H. Tuomikoski S. Kangas T. Lassi U. Kuokkanen T. Räm,ö J. (2014). Chemically activated carbon residue from biomass gasification as a sorbent for iron(II), copper(II) and nickel(II) ions. J. Water Process Eng. 4, 12–24. 10.1016/j.jwpe.2014.08.009

[B41] Serp P. Figueiredo J. L. (2009). Carbon materials for Catalysis. Hoboken, NJ: John Wiley and Sons.

[B42] Song Q. Xiao R. Li Y. Shen L. (2008). Catalytic carbon dioxide reforming of methane to synthesis gas over activated carbon catalyst. Ind. Eng. Chem. Res. 47, 4349–4357. 10.1021/ie800117a

[B43] Tavasoli A. Ozin G. (2018). Green syngas by solar dry reforming. Joule 2, 571–575. 10.1016/j.joule.2018.02.017

[B44] Trépanier M. Dalai A. K. Abatzoglou N. (2010). Synthesis of CNT-supported cobalt nanoparticle catalysts using a microemulsion technique: role of nanoparticle size on reducibility, activity and selectivity in Fischer–Tropsch reactions. Appl. Catal. A Gen. 374, 79–86. 10.1016/j.apcata.2009.11.029

[B45] Wang Z. He T. Qin J. Wu J. Li J. Zi Z. (2015). Gasification of biomass with oxygen-enriched air in a pilot scale two-stage gasifier. Fuel 150, 386–393. 10.1016/j.fuel.2015.02.056

[B46] Wigzell F. A. Jackson S. D. (2017). The genesis of supported cobalt catalysts. Appl. Petrochem. Res. 7, 9–21. 10.1007/s13203-016-0175-9

[B47] Xu L. Liu Y. Li Y. Lin Z. Ma X. Zhang Y. (2014a). Catalytic CH_4_ reforming with CO_2_ over activated carbon based catalysts. Appl. Catal. A Gen. 469, 387–397. 10.1016/j.apcata.2013.10.022

[B48] Xu L. Tang M. Liu P. Ma X. Zhang Y. Harris H. G. (2014b). Catalytic CO_2_ reforming of CH_4_ over Cr-promoted Ni/char for H_2_ production. Int. J. Hydrogen Energy 39, 10141–10153. 10.1016/j.ijhydene.2014.04.172

[B49] Yu T. Yuan Q. Lu J. Ding J. Lu Y. (2017). Thermochemical storage performances of methane reforming with carbon dioxide in tubular and semi-cavity reactors heated by a solar dish system. Appl. Energy 185, 1994–2004. 10.1016/j.apenergy.2015.10.131

[B50] Zhang G. Du Y. Xu Y. Zhang Y. (2014a). Effects of preparation methods on the properties of cobalt/carbon catalyst for methane reforming with carbon dioxide to syngas. J. Ind. Eng. Chem. 20, 1677–1683. 10.1016/j.jiec.2013.08.016

[B51] Zhang G. Su A. Du Y. Qu J. Xu Y. (2014b). Catalytic performance of activated carbon supported cobalt catalyst for CO_2_ reforming of CH_4_. J. Colloid Interface Sci. 433, 149–155. 10.1016/j.jcis.2014.06.038 25127295

[B52] Zhang G. Zhao P. Xu Y. Qu J. (2017). Characterization of Ca-promoted Co/AC catalyst for CO_2_-CH_4_ reforming to syngas production. Biochem. Pharmacol. 18, 326–334. 10.1016/j.jcou.2017.02.013

